# Whole genome duplications and expansion of the vertebrate GATA transcription factor gene family

**DOI:** 10.1186/1471-2148-9-207

**Published:** 2009-08-20

**Authors:** William Q Gillis, John St John, Bruce Bowerman, Stephan Q Schneider

**Affiliations:** 1 Institute of Molecular Biology, University of Oregon, 1229 University of Oregon, Eugene, OR 97403, USA

## Abstract

**Background:**

GATA transcription factors influence many developmental processes, including the specification of embryonic germ layers. The GATA gene family has significantly expanded in many animal lineages: whereas diverse cnidarians have only one GATA transcription factor, six GATA genes have been identified in many vertebrates, five in many insects, and eleven to thirteen in *Caenorhabditis *nematodes. All bilaterian animal genomes have at least one member each of two classes, GATA123 and GATA456.

**Results:**

We have identified one GATA123 gene and one GATA456 gene from the genomic sequence of two invertebrate deuterostomes, a cephalochordate (*Branchiostoma floridae*) and a hemichordate (*Saccoglossus kowalevskii*). We also have confirmed the presence of six GATA genes in all vertebrate genomes, as well as additional GATA genes in teleost fish. Analyses of conserved sequence motifs and of changes to the exon-intron structure, and molecular phylogenetic analyses of these deuterostome GATA genes support their origin from two ancestral deuterostome genes, one GATA 123 and one GATA456. Comparison of the conserved genomic organization across vertebrates identified eighteen paralogous gene families linked to multiple vertebrate GATA genes (GATA paralogons), providing the strongest evidence yet for expansion of vertebrate GATA gene families via genome duplication events.

**Conclusion:**

From our analysis, we infer the evolutionary birth order and relationships among vertebrate GATA transcription factors, and define their expansion via multiple rounds of whole genome duplication events. As the genomes of four independent invertebrate deuterostome lineages contain single copy GATA123 and GATA456 genes, we infer that the 0R (pre-genome duplication) invertebrate deuterostome ancestor also had two GATA genes, one of each class. Synteny analyses identify duplications of paralogous chromosomal regions (paralogons), from single ancestral vertebrate GATA123 and GATA456 chromosomes to four paralogons after the first round of vertebrate genome duplication, to seven paralogons after the second round of vertebrate genome duplication, and to fourteen paralogons after the fish-specific 3R genome duplication. The evolutionary analysis of GATA gene origins and relationships may inform understanding vertebrate GATA factor redundancies and specializations.

## Background

Most animal genomes include multiple GATA transcription factor genes with widely conserved developmental roles[1]. Within vertebrates, GATA transcription factors are required for the proper specification of cardiac and blood cell lineages, for the induction and differentiation of endoderm and mesendoderm, and in cell movement during gastrulation and neural projections. In *Xenopus laevis*, overexpression of GATA4, 5, or 6 can induce endoderm formation [2]. Similarly, the nematode GATA456 ortholog *end-1 *is necessary and sufficient to generate E or endodermal cell fate in *C. elegans*, and it also can induce endoderm when ectopically overexpressed in *Xenopus *[3].

The GATA transcription factor family is a relatively small and evolutionary tractable gene family, with only six members present in mammals, five in insects, and eleven in the nematode *C. elegans*. This gene family has undergone significant expansion in bilaterians compared to lower metazoans. For example, only a single GATA gene has been found in two cnidarian genomes currently sequenced [4].

Previous studies have demonstrated that the six vertebrate GATA factors comprise two classes of evolutionarily related genes, a GATA-1, -2, -3 class and a GATA-4, -5, -6 class [5]. These two GATA factor groups can be identified throughout bilaterian animals, suggesting that the last common ancestor of protostome and deuterostome genomes contained at least two GATA genes, with both a GATA123 and a GATA456 ortholog. Our recent survey of GATA genes from the whole-genome sequence of multiple protostome genomes has identified at least four GATA genes in every currently available protostome genome, with gene duplications having occurred only within the GATA456 class [6].

In contrast, two basal deuterostomes (invertebrate relatives of chordates), the echinoderm *Strongylocentrotus purpuratus *and the urochordate *Ciona intestinalis*, encode just two GATA transcription factor genes, similar in number to the predicted ancestral bilaterian state [5,7]. However, these GATA genes are highly divergent in sequence and bear only faint resemblance to the two GATA classes typical of most animal genomes. Indeed, a recent phylogenetic study of this gene family [8] concluded that the small GATA gene repertoire of two in *S. purpuratus *and *C. intestinalis*, relative to the eleven nematode and six vertebrate GATA genes, resulted from secondary and independent losses of GATA genes in these lineages. In addition to the uncertainty about their GATA gene origins, both echinoderms and urochordates have undergone exceptional shifts in their developmental modes relative to other deuterostome phyla. Thus it has remained difficult to ascertain the number, structural features, and roles of the ancestral deuterostome GATA gene complement.

**Figure 1:**
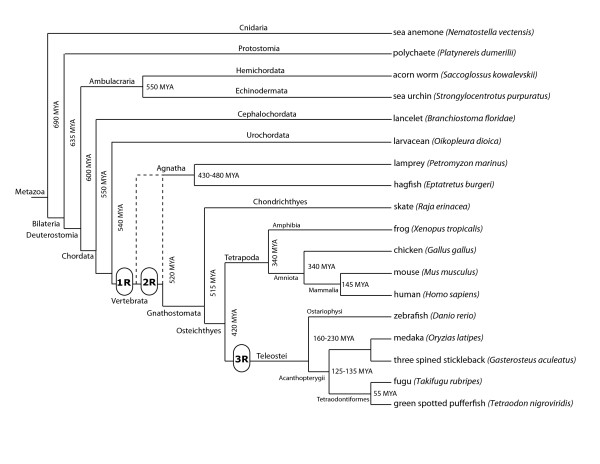
**Relationship and divergence times of deuterostome and vertebrate species**. This tree represents a survey of molecular and paleontological analyses of phylogeny and divergence times. Divergence times estimates are given in millions of years ago (MYA). The timing of genome duplication events from the first round (1R), second round (2R), and the teleost-specific third round (3R) are represented by rounded rectangles. The dotted line for the connection of the agnathan lineages represents the current uncertainty regarding their divergence relative to the second round of genome duplication.

Deuterostomes include several major groups of invertebrate and vertebrate animals (Figure 1) [9-17]. The first major deuterostome division occurred between ambulacrarians (echinoderms and hemichordates) and chordates. The chordates then split into three groups: cephalochordates, urochordates, and vertebrates. Recent studies indicate that urochordates are the closest outgroup to vertebrates [18], although urochordates are extremely diverged on both molecular and morphological levels [19]. The first split of vertebrates occurred between jawless and jawed vertebrates (agnaths and gnathostomes), followed with the divergence of jawed vertebrates into cartilaginous and bony fish (chondrichthyes and osteichtyes). There are two major groups of extant bony fish, ray-finned and lobe-finned, with the former having given rise to teleost fish and the latter to tetrapods.

There is ample evidence for multiple rounds of whole genome duplication in vertebrate lineages. Two genome duplication events are thought to have occurred near the base of the vertebrate lineages. The first genome duplication event (1R) has been proposed to occur prior to the divergence of jawed and jawless vertebrates, with a second genome duplication event (2R) occurring only in jawed vertebrates lineage [20]. However, a more recent survey of multiple lamprey and hagfish gene families concluded that the ancestor of extant jawless vertebrates also underwent two whole genome duplication events, suggesting that two rounds of whole genome duplication occurred very early in the vertebrate lineage [21]. Finally, an additional whole genome duplication event (3R) appears to have occurred in ray-finned fish [22-24]. During each of these genome duplication events, two paralogous chromosomal regions (paralogons) would be created from each pre-duplication chromosomal region, and each paralogon would initially contain a single paralog for each pre-duplicate gene. Therefore, for each 0R (pre-duplicate) deuterostome gene, there could be maximally two genome-duplicated paralogs in 1R animal genomes, four in 2R genomes, and eight in 3R genomes, though neutral drift should quickly eliminate most of duplicated paralogs [25-27]. We refer to paralogs resulting from genome duplication events as ohnologs, following the convention suggested by K. Wolfe [28] in honour of Susumu Ohno, who first proposed the occurrence of these genome duplication events during key transitions of vertebrate evolution [26,27]. Because vertebrate genomes contain six GATA factor genes, compared to only two in two different deuterostome invertebrate genomes, it has been suggested that the GATA transcription factor gene family may have expanded in vertebrates by the retention of ohnologous genes [5,7].

To more conclusively address the ancestral deuterostome condition, we have identified the GATA transcription factor complement within the whole genome sequence of two additional and less derived invertebrate deuterostomes, the hemichordate *Saccoglossus kowalevskii *and the cephalochordate *Branchiostoma floridae*. These analyses include nine diverse vertebrate genome sequences, and address gene phylogeny using both gene sequence and genomic context comparisons. Importantly, one well-conserved GATA123 gene family member and one well-conserved GATA456 family member was found within each invertebrate deuterostome genome analyzed. Thus our study provides the strongest evidence yet that the ancestral deuterostome genome contained two distinct GATA genes, one GATA123 homolog and one GATA456 homolog, from which every deuterostome GATA gene including the vertebrate complement originated. We conclude that hemichordates and cephalochordates have retained members of both GATA classes. These analyses further indicate that all vertebrate GATA genes retain conserved syntenic ohnologs, supporting the hypothesis that the expansion of the vertebrate GATA family has resulted almost exclusively from whole-genome duplication events.

## Results

### Identification of hemichordate and cephalochordate GATA sequences

While we recently concluded that the genome of the ancestor to both deuterostomes and protostomes encoded two GATA transcription factors [6,7], another group [8] suggested that at least five GATA factors were encoded by the genome of the last common ancestor of fruit flies, nematodes, and vertebrates, with subsequent losses occurring in some deuterostome lineages (see Introduction). To further address this issue, we have identified GATA factor gene sequences from the available genomes of two additional deuterostome invertebrates, the cephalochordate *Branchiostoma floridae *and the hemichordate *Saccoglossus kowalevskii*.

**Figure 2:**
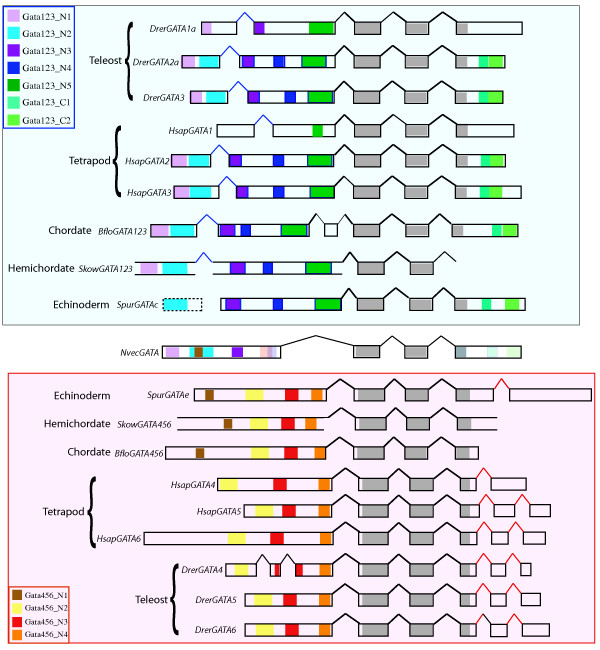
**Exon/intron structure and conserved motifs of deuterostome GATAs**. Identified exons are shown as solid blocks (boundaries confirmed by cDNA sequence) or as dotted lines (boundaries not confirmed by cDNA sequence). GATA123 orthologs for human (HsapGATA1,2,3), zebrafish (Drer1a,2a,3) hemichordate (SkowGATA123), echinoderm (SpurGATAc), and cephalochordate (BfloGATA123) are located within the blue block (top), and GATA456 (HsapGATA4,5,6, DrerGATA4,5,6, SkowGATA456, SpurGATAe, BfloGATA456) orthologs are located within the red block (bottom). The zebrafish GATA genes, Drer1b and Drer2b, are nearly identical in structure and length to Drer1a and Drer2a, respectively, and are not shown. The sole cnidarian GATA from Nematostella (NvecGATA) is shown centrally. Motifs are represented within the exons as colored blocks as specified in insets. The dotted line for the first SpurGATAc exon indicates its possible pseudo-exon status, and the open bars for SkowGATAs are due to uncertainty regarding the exact ends of the exons. Thick black lines represent ancestral eumetazoan splice sites for GATA genes, blue and red lines represent ancestral deuterostome splice sites for GATA123 and GATA456 genes, respectively, and light black lines represent novel exons.

In the cephalochordate *B. floridae *genome sequence, with 8.1× coverage, we could identify only two GATA factor genes. tBLASTn analysis of the *B. floridae *trace archives was conducted with local BLAST servers [29] to identify ~136 amino acid (AA) fragments from two distinct GATA genes. An initial reciprocal blast suggested that these fragments encode distinct GATA1/2/3 and GATA4/5/6 orthologs, and this initial assignment was also supported by the phylogenetic analyses below; therefore we refer to these as *BfloGATA123 *and *BfloGATA456*. These fragments encode a highly conserved dual zinc finger domain [5-7] within three exons (Figure 2). Two genomic scaffolds were identified from the pre-release genomic assembly JGI-assembled genome containing these fragments http://genome.jgi-psf.org/Brafl1/Brafl1.home.html. By conducting bl2seq sequence comparisons on larger regions of these scaffolds, less conserved 5' and 3' ends of each gene encoding the N-terminal and C-terminal regions of each protein was identified.

In a BLASTn search of these two predicted BfloGATA genes against sequenced EST libraries, 19 ESTs were identified for the BfloGATA123, defining the full length mRNA for this gene (1419 NT, 478 AA). Confirmation of the transcription of the BfloGATA123 gene was made by PCR amplification of a predicted 772 nucleotide (nt) fragment with gene specific primers from a gastrula/neurula cDNA library.

For the BfloGATA456 ortholog, we were unable to identify any EST from the pre-release database. We therefore defined a gene model for the 5' domain through the conserved dual zinc finger domain based on sequence comparison to human and *Platynereis *GATA sequences. Gene specific primers were designed to a predicted 5' start codon, and the conserved dual zinc finger domain. Two clones were isolated via PCR from a Bflo cDNA library, with 859 and 874 nucleotide inserts. Using the Splign program [30], these fragments both aligned to the same region of JGI:scaffold 160, and are presumably alternative splice forms. These splice forms are identical with the exception of alternative seconds exons, with the smaller splice form incorporating a novel exon that eliminates the first zinc finger domain.

In the genomic trace archive of the hemichordate *Saccoglossus kowalevskii*, with 7× coverage, we have used our Gene Family Finder program (see Methods) to computationally identify two orthologs. A reciprocal blast analysis suggested these to be a single GATA123 ortholog and a single GATA456 ortholog, which held true with the additional phylogenetic analyses below, and we have therefore named these SkowGATA123 and SkowGATA456, respectively. Through comparisons to the BfloGATA gene sequences, four exons from each SkowGATA (Figure 2) were identified. Within SkowGATA123, two exons encode the conserved first and second zinc fingers, as well as two exons 5' to the conserved zinc finger domain. No additional 3' exon sequences were identified, including the 3' conserved domain exon described for other GATAs, but it is possible that this sequence is divergent or not represented in the current trace archive. For SkowGATA456, three exons that encode the first zinc finger, second zinc finger, and a 3' conserved lysine-rich region from the conserved dual-zinc domain were identified, as well as an additional single large 5' exon.

In summary, we have found that two additional deuterostome invertebrate genomes each have only two GATA transcription factors genes, further supporting our previous conclusion that the last common ancestor to all deuterostomes had only two GATA factor genes.

### Identification of additional vertebrate GATA factors

**Figure 3:**
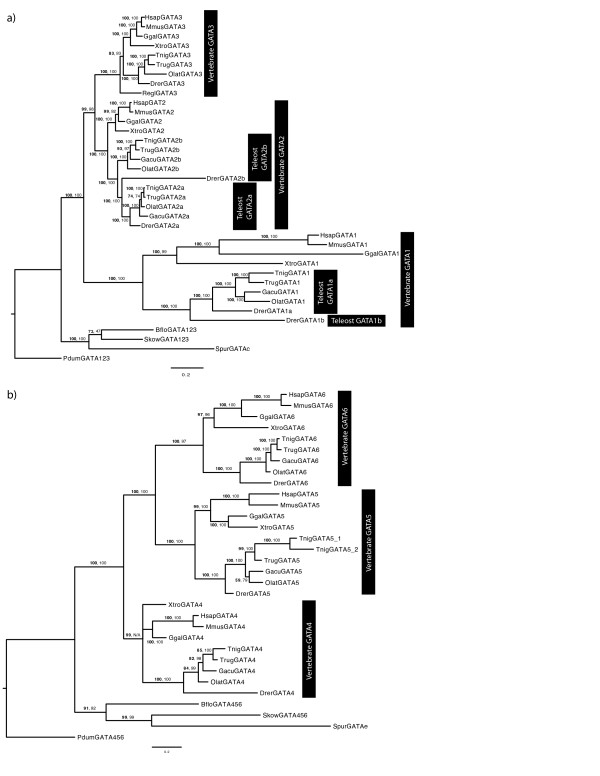
**Phylogeny of deuterostome GATA123 and GATA456 subfamilies**. Phylogenetic trees for GATA123 genes (a) and GATA456 genes (b). Branch support is given in both posterior probabilities from a Bayesian analysis (bold) or from the approximate likelihood ratio test chi-square parameter (regular). Both trees are rooted using the *Platynereis *ortholog. Species names are as follows; Bflo-*Branchiostoma floridae *(cephalochordate), Drer-*Danio rerio *(zebrafish), Ggal-*Gallus gallus *(chicken), Gacu-*Gasterosteus aculeatus *(stickleback), Hsap-*Homo sapiens *(human), Olat-*Oryzias latipes *(medaka), Skow-*Saccoglossus kowalevskii *(acorn worm), Spur-*Strongylocentrotus purpuratus *(sea urchin), Pdum-*Platynereis dumerilii *(annelid), Regl-*Raja eglanteria *(skate), Trub-*Takifugu rubripes *(fugu), Tnig-*Tetraodon nigroviridis *(tetraodon), Xtro-*Xenopus tropicalis *(frog).

**Figure 4:**
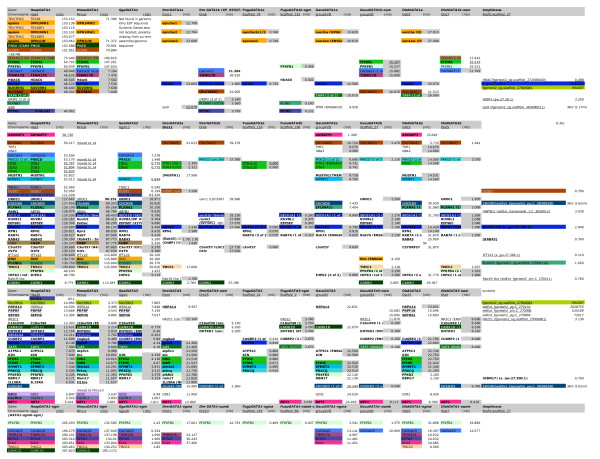
**Syntenic genes with GATA123 locus from seven vertebrate sequences**. Gene names given from ENSEMBL, location on chromosome represented in megabase. The colored blocks represent syntenic genes are part of paralogy groups syntenic with other GATA loci, following the color scheme in Figure 6.

**Figure 5:**
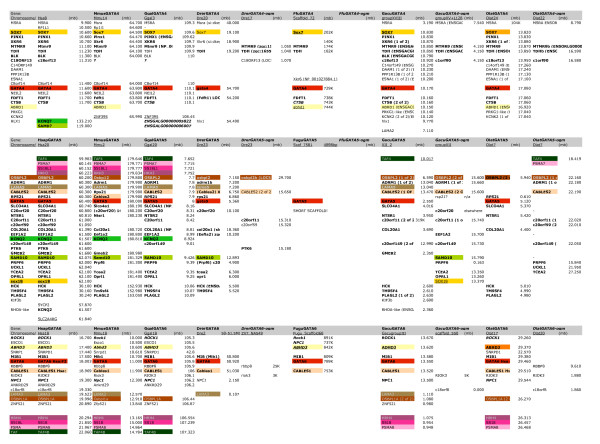
**Syntenic genes with GATA123 locus from seven vertebrate sequences**. Gene names given from ENSEMBL, location on chromosome represented in megabase. The colored blocks represent syntenic genes are part of paralogy groups syntenic with other GATA loci, following the color scheme in Figure 7.

To further investigate the expansion of GATA factors in vertebrates, we conducted exhaustive searches for GATA factor members within nine vertebrate genomes, including five teleost and four tetrapod species, again using in silico searches of annotated proteins and whole genome contigs, as well as genomic trace files, to identify the complete GATA complement for each genome. Each of the tetrapod genomes were found to contain six GATA factors genes, consistent with previous studies [5,8]. However, we also identified seven or eight GATA factor genes in each of the teleost genomes examined (Figure 3, 4, 5). The expansion from two pre-genome duplication (0R) GATA factors, to six GATA factors in the tetrapod (2R), and eight in the teleost (3R), is consistent with GATA family growth via genome duplication.

### Identification of class specific motifs

We next examined the cephalochordate and hemichordate GATA genes to determine if they include GATA123- and 456-class specific conserved coding sequence motifs, identified in a previous study [7]. BfloGATA123 exhibits one of the most complete and well conserved set of ortholog-specific motifs from our data set (Table 1 and Figure 2), containing all 7 previously identified motifs, which exhibit 38–76% amino acid identity with at least one other example of that motif. BfloGATA456 contains all 4 motifs identified within human GATA456 orthologs, and an additional N-terminal motif previously only identified in the *Platynereis *PdumGATA456, the sea urchin SpurGATAe, and the sole anemone GATA NvecGATA.

**Table 1:** Conservation of GATA motifs

	AA Percent Shared Identity															
	123_N1		123_N2		123_N3		123_N4		123_N5	Dual-ZF Domain			123_C1		123_C2	

Gene	Pd	Nv	Pd	Nv	Pd	Nv	Pd	Nv	Pd	Pd123	Pd456	Nv	Pd	Nv	Pd	Nv

BfGATA123	10	30	27	47	53	61	58	52	29	94	82	90	11	17	2	7
SkGATA123	0	14	31	50	56	61	64	64	38	77	72	74	-	-	-	-
SpGATAc	-	-	7	14	56	56	70	58	21	88	81	84	17	17	25	11
CiGATAb	-	-	10	27	7	7	44	38	21	90	81	84	11	17	33	11
HsGATA1	-	-	-	-	-	-	-	-	-	82	74	77	-	-	-	-
HsGATA2	11	18	29	50	48	33	58	64	39	92	82	86	29	23	30	12
HsGATA3	6	17	38	31	52	34	65	58	36	92	82	87	41	5	33	25
DrGATA2a	14	17	20	40	56	41	55	50	37				33	16	17	9
DrerGATA2b	5	11	17	38	32	25	55	38	29							
DrGATA3	4	24	35	27	40	26	64	58	34				35	11	30	16
DrerGATA1a	-		-	-	15	16	-	-	25							
DrerGATA1b	8	17	-	-	-	-	-	-	21							
																

	456_N1		456_N2		456_N3		456_N4			Dual-ZF Domain						

Gene	Pd	Nv	Pd	Nv	Pd	Nv	Pd			Pd123	Pd456	Nv				

BfGATA456	20	16	60	20	41	37	8			87	90	82				
SkGATA456	13	15	56	23	41	32	0			82	88	81				
SpGATAe	18	15	46	10	17	25	17			80	84	80				
CiGATAa			21	10	13	14	0			57	56	58				
HsGATA4	-	-	43	18	40	21	13			83	90	79				
HsGATA5	-	-	35	16	32	12	13			77	84	76				
HsGATA6	-	-	50	12	34	22	17			76	84	74				
DrGATA4			44	13	34	16	17									
DrGATA5			50	10	20	16	30									
DrGATA6			46	13	31	20	17									

The percent identity shared between motifs and conserved domains from cephalochordate (Bf), hemichordate (Sk), and echinoderm (Sp) GATAs compared to polychaete (Pd) and sea anemone (Nv) GATAs. Scores based upon pairwise alignment percent identity scores in individual alignments.

Similar to BfloGATA456, SkowGATA456 includes 3 N-terminal motifs identified within human GATA456 orthologs, and an additional N-terminal motif previously identified only in the *Platynereis *PdumGATATA456, the sea urchin SpurGATAe, and the sole anemone GATA NvecGATA. No SkowGATA123 conserved C-terminal motifs were detected, but all five previously identified motifs N-terminal to the zinc finger domains could be identified.

### Conserved splice site boundaries within the two deuterostome GATA classes

To further analyze deuterostome GATA gene family members, we next examined conservation of exon/intron structures. The genome assemblies were compared to the translated amino acid sequence to map splice sites and exon/intron boundaries. When the *B. floridae *cephalochordate and *S. kowalevskii *hemichordate GATA genes were compared to their human, fish, and sea anemone orthologs, we found all of the genes contain two internal introns that divide the conserved dual-zinc finger domain into first zinc finger, second zinc finger, and 3' lysine rich encoding exons (Figure 2). The positional conservation of these two introns correlates with the high conservation of the dual zinc-finger domain, relative to the rest of the protein, in almost all animal GATA transcription factors [6]. Thus, an ancestral exon/intron structure of the core conserved DNA binding domain has been retained in both deuterostome GATA123 and GATA456 gene families.

We also identified differences in the exon/intron boundaries of GATA123 and GATA456 genes, 5' and 3' of the conserved dual-zinc finger regions. These differences produce distinctive exons that encode the class specific N- and C-terminal motifs [7]. The cephalochordate BfloGata456, the hemichordate SkowGATA456, and the echinoderm GATA456 ortholog SpurGATAe, as well as the human HsapGATA4, 5, and 6 genes and the zebrafish DrerGATA5 and 6 genes, all have a single exon 5' of the zinc finger domain exons, with this one exon encoding all of the identified GATA456 motifs. However, BfloGATA123, SkowGATA123, and the human and zebrafish GATA 1, 2 and 3 genes all are encoded by two 5' exons, with two conserved motifs located within the first exon and three within the second. A comparison to the motifs shared with NvecGATA found that this intron has been observed only within bilaterian GATA123 factors [6]. We therefore suggest that the single 5' exon in GATA456 genes may represent the ancestral condition for all GATA genes, and that a subsequent intron insertion occurred shortly after the duplication of the ancestral GATA gene in the GATA123 lineage.

In contrast to their 5' exons, the 3' structure of the deuterostome GATA123 genes is more conserved than the 3' structure of the GATA456 genes. The analyzed cnidarian, echinoderm, cephalochordate, and vertebrate GATA123 orthologs each have only a single large 3' exon (though we have not yet found any 3' exons in the hemichordate SkowGATA123), and all of these orthologs contain two identifiable conserved motifs in this region. In contrast, the 3' ends of the GATA456 genes are more variable, and no conserved motifs have been identified among these genes; human and zebrafish GATA 5 and 6 genes, and the zebrafish GATA 4 gene, are split into 3 short exons, the first of which is conserved in the cephalochordate BfloGATA456. These additional two 3'-most exons appear to be diagnostic for vertebrate GATA456 genes. However, the echinoderm GATA456 ortholog has two large 3' exons, while only a single short 3' exon can be identified in the hemichordate or cephalochordate GATA456 orthologs, though these identifications may not be complete due to a lack of sequence conservation in these regions. Thus, it appears that lineage-specific acquisition of 3' exon/intron structure and sequence divergence has occurred for GATA456 factors, whereas the GATA123 factors appear to retain a 3' structure similar to that of the sole anemone NvGATA gene. Our analysis of the class-specific features, including both conserved amino-acid motifs and intron/exon boundaries, provides further support to the view that all deuterostome GATA genes are members of either the GATA123 for the GATA456 subfamilies.

### Prediction of an additional 5' exon in the *S. purpuratus *GATA123 gene (GATAc)

Because two 5' exons are conserved in chordate and hemichordate GATA genes, and because echinoderms are a sister group to the hemichordates, we were surprised that only one 5' exon was described in the previously characterized echinoderm GATA123 ortholog, SpurGATAc. Furthermore, motifs encoded by the 5' most exon in other deuterostome GATA123 genes are also present in protostome GATA123 orthologs [7], suggesting that either the *S. purpuratus *GATAc gene lost its first exon at some point, or that the gene is currently incorrectly annotated. Using tBLASTn searches of the *S. purpuratus *genomic sequence with the 5' exon from BfloGATA123, but not with those from other deuterostome GATA123 genes, we identified a 207 base pair (bp) region with significant (p = .002) similarity, approximately 18 kilobase (kb) upstream of the current exon 1 in the SpurGATAc gene. This sequence includes an open reading frame of 69 residues with an N-terminal motif 64% identical in amino acid sequence to the corresponding motif in BfloGATA123 (27% to PdumGATA123., 50% to NvecGATA123., 56% to MmusGATA2). However, this open reading frame begins abruptly within this motif and does not include a 5' start codon. This result appears to be consistent with tBLASTn searches of the *S. purpuratus *genomic trace archive directly, in which 22 of 23 traces identified containing this ORF also have a stop codon at the same position [*QVDVYYHH.], suggesting that this stop codon is not a sequencing error. Therefore, there could be an additional short 5' exon that we have not found, or perhaps this exon has degenerated and is now a pseudo-exon.

### Molecular phylogenetic analysis of deuterostome GATA genes

To better define the relationships between all deuterostome GATA factors, we conducted a series of molecular phylogenetic analyses. We first analyzed the complete set of the collected GATA factors, using the conserved zinc finger domains and aligning newly identified factors to a previously defined alignment [6]. These analyses consistently resolved GATA123 and GATA456 subfamilies (Additional File 1), but could not fully resolve relationships within the GATA123 and GATA456 clades. Alignments of GATA123 and GATA456 full length protein sequences were highly variable and generated results similar to those obtained using the conserved zinc finger domain only (data not shown).

Alignments that compared only GATA123 or only GATA456 subfamily members resulted in greater convergence of the gene tree to the species tree (Figure 3a/b). We found that invertebrate GATA123 and GATA456 genes formed separate clades outside of the vertebrate GATA123 and GATA456 clades, respectively, consistent with the 2R origin of the additional vertebrate GATAs. Within the individual vertebrate clades, there was a clear separation of tetrapod and teleost genes, and only minor changes to the species tree were observed within these groupings (compare Figure 3 to Figure 1). Outside of vertebrates, the cephalochordate GATA genes BfloGATA123 and BfloGATA456 group with the ambulacrarian orthologs (hemichordates and echinoderms), but this is likely due to the high degree of conservation and low level of divergence for both the hemichordate and cephalochordate genes.

Our results also suggest distinct ancestral relationships within each vertebrate GATA class. Within the GATA123 class (Figure 3a), a closer relationship was observed between the GATA2 and GATA3 members, to the exclusion of a more rapidly evolving GATA1 group. In contrast to previous results [5], and consistent with other recent results [8], a closer relationship between the GATA5 and 6 groups, to the exclusion of the GATA4 group, was observed within the GATA456 class.

In conclusion, these molecular phylogenetic analyses support the presence of two classes of GATA factors throughout deuterostomes. Deuterostome invertebrates possess single GATA123 and GATA456 genes, and the deuterostome GATA gene family has expanded in a manner consistent with several rounds of whole genome duplication at the base of the vertebrate lineages. See the Discussion for further consideration of these results.

### Syntenic conserved paralogs and the identification of genome-duplicated GATA paralogons

Based on the above analysis, we hypothesize that (i) the last common ancestor to all deuterostomes had one GATA123 gene and one GATA456 gene within its genome, and (ii) multiple rounds of whole genome duplication account for the expansion of vertebrate and teleost GATA genes. If this hypothesis is correct, then we should be able to detect duplicated GATA paralogons–conserved, syntenic paralogs associated with the corresponding paralogous GATA loci–within the vertebrate evolutionary lineage. To test this prediction, we characterized the adjacent genomic regions for each vertebrate GATA locus, searching for examples of tightly linked loci that have been duplicated together as a result of whole chromosome duplications. Although a superficial analysis of conserved synteny has been published [8], which describes a 'segregation' of vertebrate GATA genes on multiple chromosomes, we now describe deeper syntenies of orthologs across species and paralogs within species, and use this to completely describe the paralogons and their context during genome duplication events.

**Figure 6:**
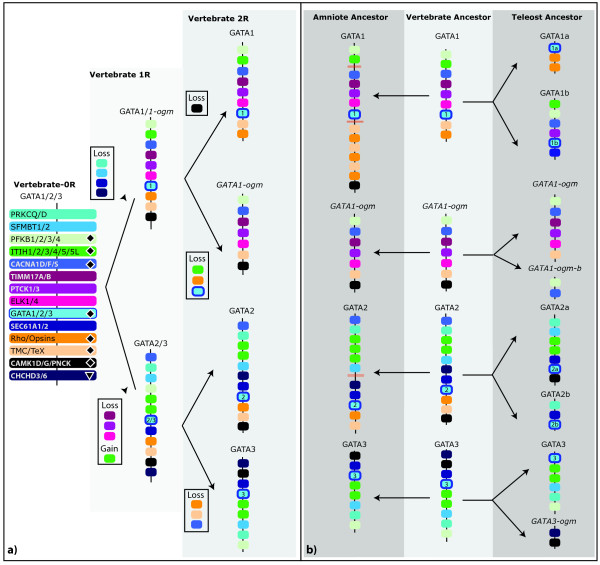
**Evolution of GATA1/1b/2/3 chromosomal regions**. Evolutionary scenario leading to the expansion of the chordate GATA123 paralogon into the four GATA1, 2, 3, and 1b paralogons during two rounds of genome duplication (a). The reconstructed GATA paralogon(s) for the vertebrate ancestor is shown after the 1R genome duplication (light grey box), or the 2R genome duplication events (medium grey box). Paralogs in the 0R vertebrate genome that can be strongly inferred when present in both the GATA1/1-ogm paralogon and the GATA2/3 paralogon (represented by diamond), or when synteny is also conserved in the cephalochordate genome (downward-pointing triangle); otherwise it is not clear if these genes were translocated independently into the 1R paralogons. Changes to the paralogons from the inferred 2R state of the last common bony fish/tetrapod ancestor (medium grey box) to the extant amniote or teleost state (dark grey box) (b). Three red bars across the chromosome indicate that a larger genomic distance separates syntenic regions on the same chromosome. Paralogous gene families include the protein kinase C (PRKCQ, D), SCM-like (SFMBT1,2), 6-phosphofructo-2-kinases (PFKFB1, PFKFB2, PFKFB3, PFKFB4), ITI heavy chains (ITIH1, ITIH2, ITIH3, ITIH4, ITIH5, ITIH5L), calcium channel subunits (CACNA1F, CACNA1D, CACNA1S), mitochondrial translocase subunit (TIMM17A, TIMM17B), PTC-kinases (PTCK1, PTCK3), ETS domain containing (ELK1, ELK2), SEC61 transport proteins (SEC61A1, SEC61A2), opsins (Rho, OPN1MW1, OPN1MW2, OPN1LW), TMC/TEX transmembrane proteins (TEX28, Z68193.2, AC092402.4, TMCC1,2), CAM-kinases (CAMK1, CAMK1D, CAMK1G, PNCK), and coiled-helix-coiled-helix genes (CHCHD3, CHCHD6).

**Figure 7:**
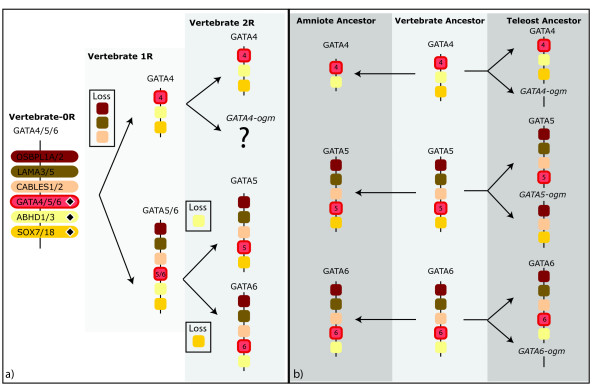
**Evolution of GATA4/5/6 chromosomal regions**. The evolutionary scenario describes losses and gains of paralogous genes near GATA456 during two rounds of genome duplication. (a) The duplications of the 0R chordate GATA456 paralogon are shown for the three GATA4, 5, and 6 paralogons (the GATA4b paralogon could not be identified). The reconstructed GATA paralogon(s) for the vertebrate ancestor is shown after the 1R genome duplication (light grey box), or the 2R genome duplication events (medium grey box). Paralogs in the 0R vertebrate genome can be strongly inferred when present in both the GATA4/4-ogm paralogon and the GATA2/3 paralogon (represented by diamond); otherwise it is not clear if these genes were translocated independently into the 1R paralogons. (b) Progression from the inferred 2R state of the last common vertebrate ancestor (medium grey box) to the extant amniote or teleost state (dark grey box).). Paralogous gene families include the Oxysterol binding like proteins, (OSBPL1A/OSBPL2), laminins (LAMA3/LAMA4), Cdk5 and Abl enzyme substrates (CABLES1/CABLES2), abhydrolase domain containing proteins (ABHD1/ABHD3), and sox transcription factors (SOX7/SOX18).

In support of GATA gene family expansion via genome duplication, we found numerous gene families with conserved synteny across the GATA loci. We first described genes syntenic with GATA123 and GATA456 loci across each of the vertebrate species (Figures 4, 5, Additional Files 2, 3 &4; see Methods). This data was used to identify gene families with paralogs syntenic in multiple GATA loci in fish and/or tetrapod species (Figures 6b, 7b). These results allowed us to define the predicted GATA paralogons within each vertebrate genome. Overall, thirteen ohnologous gene families were identified as shared between at least two of the four paralogous GATA1/2/3 regions (Figure 6a, Figure 4). Likewise five gene families are shared between the paralogous GATA 4/5/6 regions (Figures 5, 7a). Thus, all vertebrate GATA genes are located within extensive paralogons providing strong support for an origin of the vertebrate GATA gene complement by whole genome duplication events from two ancestral GATA loci, one GATA123 gene and one GATA456 gene.

By comparing the differential pattern of gene loss versus gain between the GATA paralogons within and among these vertebrate species, we infer the evolutionary birth order of the GATA paralogons by determining the most parsimonious pattern of ohnolog retention (Figures 4, 5, 6a, 7a). In this analysis, we describe clade-specific conserved losses of duplicated paralogs, though it is also formally possible that these 'losses' may represent the translocation of a pre-duplication gene into or out of a paralogon prior to a gene duplication event. Nevertheless, all cases are phylogenetically informative.

For the GATA123 family (Figure 6a), we conclude that the initial 1R duplication of the ancestral GATA123 paralogon generated a GATA1/1-ogm (ohnolog gone missing, see [31]) and a GATA2/3 paralogon, and was followed by seven subsequent paralogous gene losses. The GATA1/1-ogm paralogon lost four ohnologs, whereas the GATA2/3 paralogon lost three ohnologs (for lost ohnolog identities, see legend of Figure 6). Furthermore, within the GATA2/3 paralogon the ITIH gene apparently underwent a tandem local duplication, before the 2R duplication, resulting in the ITIH1/2/3 genes and the ITIH4/5 genes. Following the 2R duplication, the GATA1 paralogon duplicated to generate two distinct paralogons, GATA1 and GATA1-ogm (ohnolog-gone-missing, as the second GATA1 ohnolog has been lost), while the GATA2/3 paralogon gave rise to the distinct GATA2 and GATA3 paralogon. After the 2R duplication of both GATA1/1b and GATA2/3 paralogons, only seven paralogous gene losses are required to explain the inferred composition of the four resulting ancestral vertebrate GATA123 paralogons. According to our scenario, the GATA1 paralogon lost one ohnolog, the GATA1-ogm paralogon lost three ohnologs (including a second GATA1), the GATA3 paralogon lost three ohnologs, and the GATA2 paralogon lost none.

For the GATA456 family, we propose that the initial 1R duplication generated a GATA5/6 paralogon and a GATA4/4-ogm paralogon (Figure 7). This 1R duplication was followed by a severe reduction of the GATA4/4-ogm paralogon resulting in a minimum of three gene losses within this paralogon. In contrast, no gene losses occurred within the 1R GATA5/6 paralogon. We speculate, that subsequently the 2R duplication generated the GATA5 and GATA6 paralogons, and the relatively diminished GATA4 and GATA4-ogm paralogons. Due to the extensive loss of ohnologs in the latter two, we have been unable to identify a paralogous region representing the GATA4-ogm paralogon. However, our current analysis indicates that one ohnolog is missing from the GATA5 paralogon, and one ohnolog is missing from the GATA6 paralogon, with each of these two ohnologous genes being retained within the GATA4 paralogon. In contrast three pairs of ohnologs are shared between GATA5 and GATA6 paralogons.

## Discussion

### Invertebrate deuterostomes genomes encode sole GATA123 and GATA456 orthologs

To examine the evolution of GATA transcription factors in deuterostomes, including vertebrates, we searched for and identified single-copy GATA123 and GATA456 orthologs in two basal deuterostomes, the cephalochordate *Branchiostoma floridae *and the hemichordate *Saccoglossus kowalevskii*. Single-copy GATA123 and GATA456 orthologs have also been identified in two other basal deuterostomes, the echinoderm *Strongylocentrotus purpuratus *and the urochordate *Ciona intestinalis*. However, the *B. floridae *and *S. kowalevskii *genes are more conserved in sequence compared to the previously described invertebrate deuterostome GATA genes. This conservation includes near complete sets of GATA123 and GATA456 class specific sequence motifs [7], and conserved intron/exon boundaries in the gene regions that encode these motifs. Our findings confirm previous phylogenetic inferences that the genome of the last common ancestor to all deuterostomes, like the bilaterian ancestor, encoded one GATA123 and one GATA456 transcription factor, with subsequent duplications giving rise to the multiple family members present in vertebrate deuterostome genomes.

### Reconstructing the ancestral exon/intron structure and evolution of the GATA gene

By comparing the exon/intron structures of deuterostome GATA genes, we can infer the structure of the ancestral deuterostome (Ud) GATA orthologs, as well as the ancestral eumetazoan (Em) ortholog. All three of these genes contained a conserved dual-zinc finger domain encoded in three central exons, which encode the first zinc finger, second zinc finger, and a lysine-rich region. However, the 3' and 5' regions appear to vary among these genes. We infer that both the EmGATA and the UdGATA456 genes included a single 5' exon to this conserved domain, while the UdGATA123 gene contained two 5' exons. As conserved sequence motifs can be identified within the two 5' exons of the GATA123 genes, and also within the single 5'exon of the sole *Nematostella *GATA gene, we infer that the 5' region of the UdGATA123 gene gained an additional intron.

Although the 5' exon-intron structure of UdGATA456 is more similar to the NvecGATA exon/intron structure, the 3' end of GATA456 orthologs exhibits more variable features. Both the NvecGATA and the GATA123 genes terminate with the third exon of the conserved domain, while GATA456 orthologs contain a truncated third conserved domain exon as well as one or more novel 3' exon(s). This third-conserved domain exon encodes ~27 conserved amino acids of a lysine rich region. However, the GATA123 and NvecGATA genes encode a less-conserved terminal end with two C-terminal motifs. In comparison, this lysine-rich exon is shorter in GATA456 orthologs than in the GATA123 or NvecGATA genes, and lacks C-terminal sequence motifs. Furthermore, GATA456 genes contain novel exon(s) 3' to the conserved domain, and we have been unable to identify conserved motifs from this additional region, suggesting that the 3' region has undergone significant evolutionary change in GATA456 paralogs.

All vertebrate GATA456 genes have lost the ancestral N-terminal motif N1, which is present within deuterostome invertebrate GATA456s, and within the protostome annelid *Platynereis dumerilii *GATA456 ortholog. A BLAST search of the human N-terminal region against the NR protein database fails to find this motif in any vertebrate GATA transcription factor, suggesting that this motif may have been lost early in vertebrate evolution.

### Greater sequence conservation of GATA123 orthologs

Comparison of different deuterostome GATA genes to the sole cnidarian GATA (NvecGATA) gene also suggests that GATA123 genes are more slowly evolving then their GATA456 counterparts. This can be seen both in the higher percent identity shared between the conserved domains of the deuterostome GATA123 and NvecGATA genes (Table 1), the high affinity of the BfloGATA123 with the NvecGATA, and the total number of common motifs we can identify. Perhaps GATA123 genes are more constrained due to their retention of a deep ancestral function, while the GATA456 class might be more diverged due to the selection or incorporation of bilaterian or phylum specific roles. This view is consistent with previous comparisons of the GATA gene complement in multiple protostome genomes. Almost all protostomes possess a single copy, more slowly evolving GATA123 gene, whereas the GATA456 genes expanded in many protostomes by sequential tandem duplications and subsequent modifications to their gene structure [6]. However, the expression patterns currently described for deuterostome and cnidarian GATA factors are not consistent with retention of a deep ancestral function within the GATA123 class. Whereas NvecGATA mRNA is largely restricted to the endoderm in the cnidarian *Nematostella *[4], with only a small ectodermal expression domain, the vertebrate GATA-1, -2, and -3 are expressed and function mostly within ectodermal tissues and blood, but not in the endoderm [1]. However, GATA gene expression has not been examined in many cnidarian species, and thus any inference of any ancestral GATA function deeper in animal phylogeny than bilaterians is still premature.

### Expansion of vertebrate GATA transcription factor genes during two rounds of whole genome duplications

Our previous work and this analysis suggest that the last common ancestor to both protostomes and deuterostomes had single GATA123 and GATA456 genes. But these two GATA classes have undergone distinct expansions using different mechanisms during the subsequent evolution of different animal phyla. In protostomes, only the GATA456 class appears to have undergone expansion, at least in part by tandem duplications within individual chromosomes. By contrast, in vertebrates, both the GATA123 and the GATA456 family have expanded through the retention of duplicated GATA genes that originated during two rounds of whole genome duplication [32]. Our molecular phylogenetic analysis, and our analysis of conserved syntenic paralogs, both support expansion by whole genome duplication and furthermore suggest a specific evolutionary order for these duplication events (compare scenarios in Figure 6a and 7a to the clades defined in Figure 2).

Our molecular phylogenetic analysis of GATA123 genes (Figure 3a) reveals a closer relationship between GATA2 and GATA3 orthologs, to the exclusion of a more rapidly evolving GATA1 group. It is not surprising that the GATA2 and GATA3 genes show more affinity to each other, as GATA1 appears to be a fast-evolving ortholog relative to other vertebrate GATAs. Nevertheless, these relationships are further supported by the retention of more syntenic paralogs between the GATA2 and GATA3 loci, then between the GATA1 and either GATA2 or GATA3 loci. However, the conservation of syntenic paralogs between GATA1 and either GATA2 or GATA3 strongly supports common evolutionary origin of all three from an ancestral GATA123 paralogon. We therefore conclude that GATA2/3 and GATA1 intermediates were generated after the 1R vertebrate genome duplication, and that one GATA1 ohnolog (chromosomally-duplicated paralog) was lost after the 2R duplication.

In contrast to previous results [5] but consistent with other more recent results [8], our molecular phylogenetic analysis suggests a closer relationship between the GATA5 and 6 groups, to the exclusion of the GATA4 group. However, despite the differing outcomes in previous molecular phylogenetic analyses, our synteny analysis is consistent with our phylogenetic analysis, and further supports a closer relationship between the GATA5 and 6 groups. We conclude that the 1R genome duplication produced GATA4 and GATA5/6 intermediates, and an additional GATA4-ohnolog had been lost after the 2R genome duplication.

### Additional gene duplications in teleosts

We also find evidence for additional teleost-specific ohnologs in the GATA123 lineage, but not in the GATA456 lineage corresponding to an additional round of genome duplication at the base of teleost fish. This evidence stems from both overlaying the species phylogeny on the gene phylogeny (compare Figure 1 to Figure 3) to find additional 3R duplicates, but more conclusively from the comparisons of duplicated paralogons. In all, four additional teleost paralogs have been identified, and two of these paralogs clearly resulting from larger chromosomal duplications.

The topology of our molecular phylogenetic analysis (Figure 3a) indicates a teleost-wide duplication of the GATA2 gene into separate GATA2a and GATA2b genes, most likely originating from the 3R teleost-specific genome duplication event. Although the topology of zebrafish GATA2b within the tree is slightly off, possibly due to its long branch indicating its derived sequence, the presence of conserved syntenic genes between the tetrapod GATA2 paralogon and the teleost GATA2a and GATA2b paralogons strongly suggests that these duplicated via a chromosomal duplication.

The zebrafish genome also contains a second GATA1 duplicate that appears to originate from the 3R duplication. Although only found in zebrafish, our phylogenetic analysis suggests that this GATA gene may have resulted from the 3R duplication, and was secondarily lost early in the ancestor of all other teleost. This view is consistent with zebrafish being the most basal member of the fish species represented in this analysis. However, this view is also supported by the presence of two identifiable GATA1 paralogons within each teleost fish genomes (Figure 4, Figure 6b, Additional File 4), although the second GATA1 gene is missing in these additional teleost paralogons with the exception of zebrafish.

Although we see no evidence for additional teleost GATA456 ohnologs from a 3R round of genome duplication, the *Tetraodon *(green spotted pufferfish) genome does contain two GATA5 paralogs. However, the topology of our molecular phylogenetic analysis suggests a more recent origin via a *Tetraodon*-specific gene duplication, as opposed to retained genome duplicate.

Our analysis of conserved synteny demonstrates the presence of additional duplicated paralogons, even when a second GATA paralog is not identified (see Additional File 4 for complete Discussion). Therefore, our data based on the comparative analysis of GATA paralogons in vertebrates strongly supports a third genome duplication event (3R) at the base of teleost fish.

It is notable that so many (6/8) of these GATA transcription factors were retained after the first two rounds of genome duplication on the base of the vertebrate branch. In comparison, a recent analysis from the cephalochordate genome could identify retention of genome duplicated paralogs in only about one quarter of all human gene families, with a much smaller fraction containing multiple ohnologs [33]. Furthermore, only 2/6 ohnologs (GATA1a/1b, zebrafish GATA2a/2b) were retained after an additional teleost-specific whole genome duplication event. Apparently, the integration and preservation of GATA transcription factors into the gene regulatory networks was a more probable outcome after the two early rounds of whole genome duplication (1R and 2R), and less likely after the third round (3R). In addition, after these early genome duplications at the base of the vertebrate lineage, the GATA gene family has remained static in most vertebrate species. After the 2R duplication event, all of the examined tetrapods maintained exactly six GATA transcription factor genes. After the teleost-specific 3R genome duplication, only a single gain of a GATA5 duplicate in *Tetraodon*, and a loss of the GATA2b ohnolog in the ancestor of the acanthopterygian fish, has occurred.

The presence of two distinct GATA factor classes in basal deuterostomes, and their subsequent expansions in vertebrates, informs the understanding of studies indicating functional redundancy within each GATA class. For example interfering with the function of all three GATA456 orthologs in *Xenopus laevis *embryos results in a much more severe endoderm defect than does an inhibition of the function of only one or two of them. Similarly, reducing the function of only one or two GATA456 paralogs only partially blocks cardiac mesoderm induction in both zebrafish and *Xenopus *[2,34,35]. The overlapping expression domains in the CNS for the GATA2 and 3 [36] and in hematopoietic lineages for GATA123 orthologs [1] may suggest that these GATA factors also have redundant functions. Similarly, the GATA123 and GATA456 gene families in nematodes are both highly redundant in their requirements. The use of *C. elegans *GATA456 gene duplicates at multiple nodes and levels in an endodermal gene regulatory network provides the furthest understood model so far for retention, cooption, and integration of gene duplicates within a gene network over evolutionary time [37].

While these gene expansions and functional redundancies can complicate studies of GATA functions, both the hemichordate and the cephalochordate posses only single copies of each GATA factor class. Both of these basal deuterostomes also exhibit many widely conserved morphological features that are thought to resemble the ancestral states for both deuterostomes and chordates, respectively (reviewed in [38-41]). Thus these basal deuterostomes are appealing model organisms in which to investigate conserved functions for the GATA123 and GATA456 classes of developmental regulatory transcription factors.

## Conclusion

The above molecular phylogenetic analyses, as well as comparisons of conserved intron/exon structure and sequence motifs, demonstrates that the last common ancestor to all deuterostomes had only two GATA factor genes, one GATA123 and one GATA456 gene, within its genome. These analyses confirm that the GATA family of transcription factors has expanded via whole genome duplications in vertebrates. During the 1R and 2R genome duplication, this family expanded to three GATA123 and three GATA456 genes that are conserved across vertebrates. The 1R genome duplication gave rise to GATA1 and GATA2/3 paralogs, as well as GATA4 and GATA5/6 paralogs, while single GATA ohnologs were lost from the GATA1 and GATA4 lineages after the 2R event. In addition, the teleost 3R genome duplication has resulted in 1 or 2 additional GATA123 duplicates in fish species. Both, our molecular phylogenetic analysis and conserved paralogon analysis are in support of the same birth order and relationships between GATA123 and GATA456 subfamilies members.

The identification of single GATA123 and single GATA456 orthologs in the more conserved hemichordate and cephalochordate genomes highlights how these basal deuterostomes may provide useful model systems for investigating conserved GATA factor requirements without the functional redundancies observed in vertebrates. Additionally, although this paper focuses on GATA factor gene relationships, our analysis has also identified eighteen other gene families contained within paralogous gene groups. The reconstructed evolutionary history of these vertebrate GATA paralogons provides a new basis for understanding the origin and evolution of these paralogous gene families as well.

## Methods

### Identification of *Branchiostoma floridae *GATA sequences

Initial identification of two GATA gene fragments was conducted using tBLASTn analysis of the *B. floridae *trace archive. These fragments were used to search for the chromosomal regions containing these sequences in the draft genome (1.0) of *B. floridae*. BfloGATA123 was found on JGI_Scaffold27 (2842113–2842359), as well as an allelic copy on JGI_Scaffold 383; in the newer version (2.0), only one BfloGATA123 containing contig was found (on Bf_V2_141). BfloGATA456 was identified on JGI_Scaffold160 (117014–51465), as well as an additional allelic copy on Scaffold_714; two allelic contigs were also found in the second assembly (on Bf_V2_327 and Bf_V2_265. Additional sequence on the 5' and 3' ends were identified via BLAST 2 Sequences (bl2seq) [42] comparisons against GATA123 and GATA456 orthologs from *Platynereis, S.purpuratus*, and vertebrate GATA sequences. We also identified 19 expressed sequence tags (ESTs) for the single BfloGATA123 gene, which allowed a precise definition of the full-length coding sequence of this gene (1419 NT, 478AA). No published ESTs were available for the predicted GATA456 sequence.

We amplified by PCR these two GATA fragments from a *B. floridae *gastrula-neurula stage cDNA library, generously provided by James Langeland [43]. For BfloGAT456, we isolated two different sized clones (859 nt and 874) using the following primers: F1BfG456 (BfloGATA456-'MYQ') 5'-ATGTACCAGAATCACTCCGTCGCG-3'; R1BfG456 (BfloGATA456- '3'aln') 5'-ATTACTGGTGCTAGTTGGAGGCTTGC-3, designed to conserved regions from the 5' and 3' regions based on our in silico predictions. These fragments appear to be alternate splice forms, with the smaller clone (BfloGATA456-isoform b) encoding an alternative second exon, resulting in the loss of the first zinc finger in this isoform.

For BfloGATA123 PCR amplified a 772 base pair fragment (corresponding to nt129–1070 of the published BfloGATA123 cDNA) using nested gene specific primers F1BfG123 (BfGATA123 – F129) 5'-AGACATCGACGTGTTCTTCCACCA-3'; F2BfG123 (BfGATA123 – F300) 5'-CATGCAGTGGATCGAGAGTACCAA-3'; R1BfG123 (BfGATA123-R1128) 5'-TGTCTGGATGCCGTCCTTCTTCAT-3' R2BfG123 (BfGATA123-R1070) 5'-TAAAGTCCACAGGCGTTGCACACA-3').

### Identification of Saccoglossus *kowalevskii *GATA sequences

A bioinformatics pipeline, Gene Family Finder (GFF), was developed to facilitate the identification of gene-family members within genomic trace archives, and used to search for GATA genes from the hemichordate *Saccoglossus kowalevskii *(Additional File 5). This tool takes a user protein sequence, and compares this to a local genomic trace DNA BLAST database using protein-translated nucleotide (tBLASTn) comparison. The program then clusters all the initial hits into unique groups of redundant traces by taking into account the greater divergence between nucleic acid sequences relative to amino acid sequence. Our program iterates through the initial hit list, performing a BLASTn search on the hit identifying overlapping traces based upon an e-value cut off. These overlapping traces are then collected into their own unique hit file, and used to remove these traces from the initial results list. These unique-hit redundant traces are assembled into a larger DNA contig using the CAP3 program [44]. These contigs are compared to the input sequence using the bl2seq program, and these results are parsed to display both the aligned region, and translation(s) of the contig based upon significant bl2seq identified frames. To test the utility of this program, we compared the results of previous analyses [6], and were able to identify conserved open reading frames (ORFs) for the complete GATA gene complement in various protostome species. In addition, we confirmed the utility of GFF to identify very divergent GATA sequences in whole genome trace archives of the urochordate *Oikopleura dioica*, that exhibits one of the most divergent invertebrate deuterostome genome sequences. GFF was able to identify two divergent GATA gene family members orthologous to the GATA gene complement found in two urochordates *C.intestinalis *and *C.savingii *(data not shown). Searching the current trace archive of *S.kowalevskii *from the NCBI trace archive (8,246,246 sequences; last updated April 9, 2008) we identified a total of 8 ORFs that belong to a sole SkowGATA123, and a sole SkowGATA456 gene.

### Identification of vertebrate GATA sequences

Protein sequences were collected from ENSEMBL database (v52) for four tetrapods, frog (Xtra-*Xenopus tropicalis*), chicken (Ggal-*Gallus gallus*), mouse (Mmus- *Mus musculus*), and human (Hsap – *Homo sapiens*), as well as 5 teleost species, zebrafish (Drer – *Danio rerio*), medaka (Olat – *Oryzias latipes*), stickleback (Gacu – *Gasterosteus aculeatus*), fugu (Trub – *Takifugu rubripes*), and tetraodon (Tnig – *Tetraodon nigroviridis*), and was combined with our own annotated sequences for a hemichordate (Skow – *Saccoglossus kowalevskii*), lancelet (Bflo – *Branchiostoma floridae*) and a polychaete (Pdum – *Platynereis dumerilii*). In cases where a single gene encoded multiple transcripts, we selected the protein that appeared to be most complete, e.g. most closely followed the ancestral intron/exon pattern (described below). Each of the vertebrate genomes was searched again using tBLASTn analyses, to add additional unannotated GATA factors from these genomes (primarily from fish species). We used our gene family finder program to further probe the zebrafish genome (which could identify all eight zebrafish GATA factors). Additional sequences were collected from the NCBI protein database for single GATA factors isolated from the hagfish (Ebur – *Eptatretus burgeri*) and skate (Regl – *Raja eglanteria*), and for the previously identified chicken GATA1 cDNA sequence. The chicken GATA1-cDNA appears to be missing in the current chicken assembled genome, and cannot be identified via tBLASTn searches of the genomic trace sequence, along with many other genes syntenic with this region of human and mouse chromosome X. The lack of this entire chromosomal regions, but the presence of a chicken GATA1-cDNA sequence and other cDNAs syntenic with the GATA1-paralogon (see Additional File 4), suggests that this region may have been missed during sequencing of the chicken genome.

### Phylogenetic analysis

Protein sequences from each vertebrate and invertebrate deuterostome genome (excluding the highly divergent Urochordate genes) were aligned using MUSCLE [45], and an initial round of phylogenetic analysis (data not shown) was used to divide the sequences into either GATA123 or GATA456 transcription factors. These files were then re-aligned using MUSCLE to improve subfamily alignments.

Topology of the phylogenetic trees were generated from a Bayesian analysis with MrBayes (version 3.1 parallel, on an eight processor linux system) [46], using the Gamma rate parameter and the WAG model, and is based upon the consensus tree of two converged runs of 3,000,000 generations using 4 chains, burnin of 500,000 generations; branch support represent posterior probabilities. A maximum-likelihood phylogenetic analysis was conducted using PHYML-alrt (v2.4.4) [47,48], using the WAG model, 4 substitution rate categories, and maximum-likelihood estimates for the gamma distribution parameters and proportion of invariable sites. Branch support is given via the approximate likelihood test Chi-square based parametric branch supports.

### Motif and splice site analysis

GATA123 and GATA456 motifs outside of the conserved dual-zinc finger domain were identified as described previously [7], and were manually aligned to the *S. kowalevskii *and *B. floridae *orthologs. A motif was identified if it shared at least a 20% pairwise identity with another example of that motif. Splice boundaries were identified by using the Splign program [30].

### Synteny analysis

To examine the GATA genomic microenvironment, we identified genes syntenic with 6 GATA loci across chicken, mouse, and human (amniote) chromosomes. This was done using the ENSEMBL genome browser (release 52), selecting the ContigView for each of the 6 human GATA loci, and then using the view syntenic location option with either chicken (*Gallus gallus*) or mouse (*Mus musculus*). As the gene order was largely consistent across all three amniote vertebrates, an ancestral amniote chromosomal region for each of the six GATA loci was based upon their order first in the human genome, and then by their location in mouse or chicken (if absent from human); however, using chicken or mouse first results in a very similar gene order suggesting that all of three species largely retained their ancestral synteny for this region.

We then proceeded to identify orthologs of genes syntenic around the human GATA loci from three fish species; zebrafish, medaka, and stickleback, using a reciprocal best BLAST hit (RBH) prediction database [29]. Additionally, we used the ENSEMBL BioMart (V52), starting with genes from the human chromosomal regions identified above, to collect human paralogy annotations as well as orthology annotations in seven analyzed vertebrate genomes. Similar analyses were conducted to compare the 6 human and 12 zebrafish GATA loci to current *Branchiostoma *genome assemblies (V1.0 and V2.0), though only a limited number of syntenic genes were identified between vertebrates and cephalochordates.

## Authors' contributions

BQG and JSJ performed sequence and evolutionary analyses, and wrote the gene family finder code. BQG and SQS designed the study and analyzed the data. BB and SQS conceived and supervised the study. BQG, BB, and SQS drafted the manuscript. All the authors read and approved the final manuscript.

## Supplementary Material

Additional file 1 **Molecular Phylogeney of all deuterostome GATA genes**. Newly identified deuterostome GATAs were aligned to each other using MUSCLE, and then the conserved domain was manually trimmed to the conserved domain and aligned as a block to the conserved domain from a previous analysis [6]. This alignment was used for maximum-likelihood molecular phylogenetic analyses. This tree was generated using PHYML-alrt using the WAG model of evolution and 4 substitution rate categories, and branch support given as chi-square proportions, and midpoint rooted. Click here for file

Additional file 2 **Table of GATA123 syntenic genes**. ENSEMBL Gene IDs, chromosomal location, and gene start is given for the human gene for each gene included in the GATA123 paralogous region, as well as the human paralog, annotations, and orthologs annotations in seven vertebrate species. Click here for file

Additional file 3 **Table of GATA456 syntenic genes**. ENSEMBL Gene IDs, chromosomal location, and gene start is given for the human gene for each gene included in the GATA456 region, as well as the human paralog, annotations, and orthologs annotations in seven vertebrate species. Click here for file

Additional file 4 **Ancestral and extant GATA paralogons**. Complete discussion of GATA chromosomal evolution. Click here for file

Additional file 5 **Gene Family Finder (GFF)**. Outline of the Gene Family Finder (GFF) program as a flowchart. Click here for file
