# Supplementary figures and images for: Whole genome duplications and expansion of the vertebrate GATA transcription factor gene family
Source: BMC Evol Biol. 2009 Aug 20;9:207. doi: 10.1186/1471-2148-9-207 (PMC2857956; doi:10.1186/1471-2148-9-207)

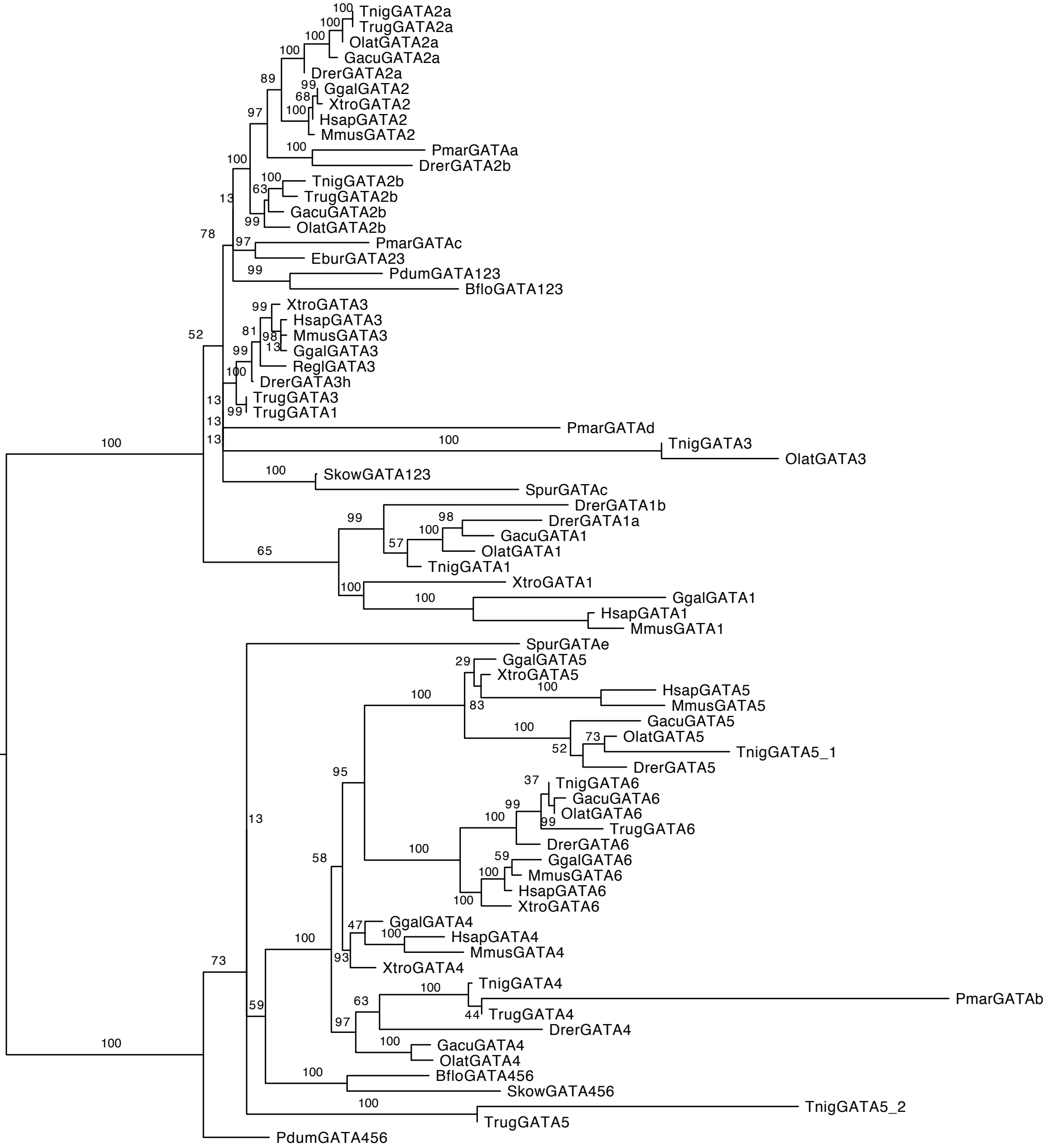

Supplement: Additional file 1 — Molecular Phylogeney of all deuterostome GATA genes. Newly identified deuterostome GATAs were aligned to each other using MUSCLE, and then the conserved domain was manually trimmed to the conserved domain and aligned as a block to the conserved domain from a previous analysis [6]. This alignment was used for maximum-likelihood molecular phylogenetic analyses. This tree was generated using PHYML-alrt using the WAG model of evolution and 4 substitution rate categories, and branch support given as chi-square proportions, and midpoint rooted. [file 1471-2148-9-207-S1.pdf]

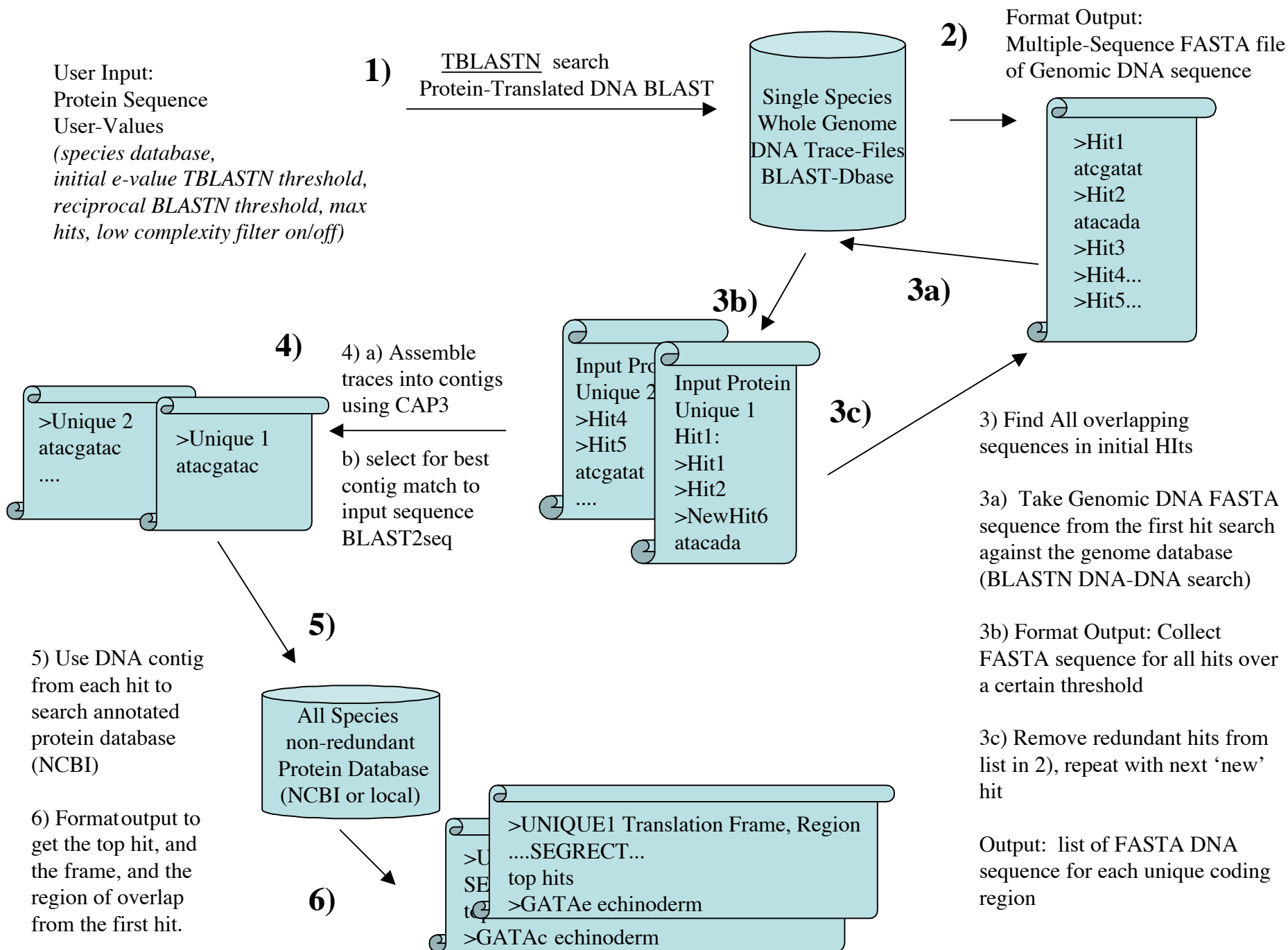

Supplement: Additional file 5 — Gene Family Finder (GFF). Outline of the Gene Family Finder (GFF) program as a flowchart. [file 1471-2148-9-207-S5.pdf]
