# Supplementary material for: Whole genome duplications and expansion of the vertebrate GATA transcription factor gene family
Source: BMC Evol Biol. 2009 Aug 20;9:207. doi: 10.1186/1471-2148-9-207 (PMC2857956; doi:10.1186/1471-2148-9-207)
Supplement: Additional file 4 — Ancestral and extant GATA paralogons. Complete discussion of GATA chromosomal evolution. [file 1471-2148-9-207-S4.doc]

Additional File 4: Additional description of ancestral and extant GATA paralogons

**1.The GATA123 paralogon**

1.1. The ancestral vertebrate GATA123 paralogon (pre R1)

Based upon our analysis, we would predict that one ancestral gene from the following gene families were likely syntenic in the ancestral GATA123 locus (pre-first round genome duplication); the protein kinase C (PRKCQ,D), SCM-like (SFMBT1,2), 6-phosphofructo-2-kinases (PFKFB1,2,3,4), ITI heavy chains (ITIH1,2,3,4,5,5L), calcium channel subunits (CACNA1F,D,S), mitochondrial translocase subunit (TIMM17A,B), PTC-kinases (PTCK1,3), ETS domain containing (ELK1,2), SEC61 transport proteins (SEC61A1,2), opsins (Rho, OPN1MW1,MW2,LW), Transmembrane and coiled-coil domains proteins (TEX28, TEX28P1, TEX28P2, TMCC1,2), CAM-kinases (CAMK1,1D,1G, PNCK), and coiled-helix-coiled-helix genes (CHCHD3,6). We have also discussed below several paralog/ortholog groups that retained conserved synteny across species, when additional data is needed to support a paralogon.

1.2. The ancestral vertebrate GATA1/1-ogm and GATA2/3 paralogons (pre R2)

After the first round of genome duplication two GATA123 paralogons were generated from the initial GATA123 chromosome, which we infer to be the GATA1/1-ogm paralogon, and the GATA2/3 paralogon. We infer these paralogons would then have undergone a total of 7 losses from these paralogons. From the ancestral GATA123 chromosome, the GATA1/1-ogm paralogon would have lost the *PRKCQ/D*, SFMBT1/2, SEC61A1/2, and CHCHD3/6 ohnologs. After these loses, the GATA1/1-ogm paralogon would be left with NET1, CACNA1F/S, TIMM17A/B, PCTK1/3, ELK1/4, Rho/Opsins, TMCC2/Tex28, GDI1, PFKFB1/2, AKT3 (PRKC), ITH5L, HDAC6, SLC38A5, ZCCHC13 (CNBP), GATA1, PLXNA2/3, PNCK/CAMK1G, and SUV39H1.

Meanwhile, the GATA2/3 paralogon would have lost TIMM17A/B, ELK1/4, and PTCK1/3 ohnologs, and also appears to have undergone a duplication of the ITIH gene, resulting in the ITIH1/2 genes and the ITIH3/4/5 genes. Therefore the GATA2/3 paralogon would contain SUV39H2, SEC61A1/2, CACNA1D, PRKCQ/D, SFMBT1/2, PFKFB3/4, ITIH1/2, ITIH3/4/5, NET1/ARHGEF3, Rho/Opsins, TMCC1, CAMK1/K1D, RBM17, GDI2, CALML5/TNNC1, and SLC38A3 orthologs.

1.3. The ancestral vertebrate GATA1, 1-ogm, 2, and 3 paralogons (post R2)

After the second round of genome duplication, four GATA123 paralogons were generated, including GATA1, GATA1-ogm (ohnolog gone missing, which has lost the duplicate GATA1 paralog), GATA2, and GATA3. Only seven more losses are required to explain this next round of paralogon duplications.

The GATA1 paralogon lost the CAMK1D and AKT3 ohnolog, and has also undergone three tandem duplications of the TEX28/Opsin genes, resulting in PFKFB1, ITH5L, CACNA1F, TIMM17B, HDAC6, PCTK1, SLC38A5, ELK1, GATA1, SUV39H1, TEX28/28P1/28P2, OPN1MW2/MW1/LW, PLXNA3, PNCK, GDI1, ZCCHC13 (CNBP).

The GATA1-ogm paralogon lost the GATA1-duplicate, and two other paralogs (TMC/TEX and the ITIH5L), leaving it with PFKB2, CACNA1S, TIMM17A, PCTK3, ELK4, TMCC2, and CAMK1G.

We did not identify clear losses of paralogs in the GATA2 paralogon, and the GATA2 paralogon retained ARHGEF3, CACNA1D, TNNC1, PFKFB4, PRKCD, SFMBT1, ITIH1, ITIH3, SLC38A3, GATA2, CNBP, RHO, TMCC1, IFT22, SEC61A1, PLXNA1, CHCHD6, and CAMK1 orthologs.

The GATA3 paralogon lost Opsin, TMCC1 and CACNA1 ohnologs, and retained CAMK1D, SEC61A2, GATA3, ITIH2, ITIH5, SFMBT2, PRKCQ, PFKB3, RBM17, GDI2, CALML3, and NET1 orthologs.

1.4. The ancestral amniote GATA1, 1-ogm, 2, and 3 paralogons

We infer the ancestral amniote GATA paralogons from comparisons of chicken, mouse, and human genomic regions. In comparison to fish, the amniote paralogons appear largely intact with most syntenic genes retained from the vertebrate ancestor, but have undergone some large chromosomal translocations.

For the GATA1 paralogon, there appears to be at least one (and likely two) large intrachromosomal translocations that can be revealed by comparisons to the fish paralogons, and the GATA2 and GATA3 paralogons. These result in a block of genes containing the TEX28, OPN1MW2, TEX28P1, OPN1MW1, TEX28P1 OPN1LW, PNCK, DUSP9, separated by a large genomic interval from a distinct ZCCHC13 homolog, which is also separated by a large genomic interval from another block of genes containing ITIHL, PFKFB1, CACNA1F, TIMM17B, HDAC6, GATA1, SUV39H1, SLC38A5, and PTCK1. Also, it appears that the vertebrate TEX28/OPN1 genes have undergone two tandem duplication, resulting in three additional paralogs of either type.

For the GATA2 paralogon, there appears to be at least one large intrachromosomal translocation, which is revealed by comparisons to the fish paralogons and the GATA3 paralogon. The first block of genes contains, ARHGEF4, CACNA1D, PRKCD, ITIH1, ITIH3/4, SFMBT1, TNNC1, DUSP7, SLC38A3, and PFKFB3, separated by a large genomic space from a second block of genes which contain linked CHCHD6, PLXNA1, SEC61A1, GATA2, CNBP, RHO, PLXND1, TMCC1 orthologs.

The GATA3 paralogon appears relatively intact (though not in a very gene-dense chromosomal region). The SUV39H2, CAMK1D, SEC61A2, GATA3, ITIH2, ITIH5, SFMBT2, PRKCQ, PFKB3, RBM17, GDI2, CALML3, and NET1 orthologs are relatively closely linked, whereas PTCK1 is syntenic but separated by a large genomic distance.

1.5. Modification to GATA paralogons in extant tetrapod species (chicken, mouse, human)

From the ancestral amniote condition, we can identify very few losses/changes among amniotes. However, one glaring omission is the absence of the complete GATA1 paralogon in the current chicken genome assembly (ensembl V52), or of the GATA1 from WGS trace sequence (see methods). However, a chicken GATA1 EST has been cloned, as well as ESTs for other chicken genes in the GATA1 paralogon. Mice and humans appear to have largely similar GATA1 paralogons both on their X chromosomes, although similar to fish the TEX28 coiled-coiled domain proteins have not been identified.

The GATA2 paralogon in mice appears to have translocated the first block of genes containing ARHGEF4, CACNA1D, PRKCD, ITIH1, ITIH3, SFMBT1, TNNC1, DUSP7, SLC38A3, and PFKFB3 to chromosome 14. Humans appear to have an additional ITIH3 gene (ITIH4), whereas chicken appears to be missing or have translocated ARHGEF3 and SLC38A3 orthologs.

The GATA3 paralogon appears to be largely similar across humans, mice, and chickens. The largest difference in the GATA3 paralogons is the transfer of a block of paralogs (GDI2, CALML3, and NET1) in mice that are found on chromosome 13, whereas the remainder of the GATA3 paralogon is on chromosome 2.

1.6. The ancestral fish GATA paralogons

As fish appear to have undergone a third whole round of genome duplication, for each ancestral chromosome we would predict to find two additional paralogons each. If this scenario is correct we would expect two fish paralogons for each the GATA1, GATA1-ogm, GATA2, and GATA3 containing paralogons present in the R2 ancestor, giving a total of eight teleost GATA123 paralogons.

We have inferred eight GATA paralogons that existed prior to the divergence of acanthopterygiian and ostariophysian fish, based upon our comparisons of five extant species (see below). Relative to amniotes, the fish paralogons appear to have lost paralogs more extensively, and therefore generally contain fewer conserved paralogs; yet for the most part their identification of two paralogons is fairly straight forward.

The GATA1 paralogon gave rise to both GATA1a and GATA1b paralogons in teleost fish. The GATA1a paralogon appears to be greatly reduced, but can be identified based upon the presence of two opsin duplicates (Opn1lw, Opn1sw), GATA1a, and TAF4. The GATA1b paralogon, on the other hand, would contain ITIH5l, PFKFB1, CACNA1F, TIMM17B, GATA1b, and SLC38A5, PLXNB3, and PCTK1 orthologs.

The GATA 1-ogm paralogon gave rise to both GATA1-ogm-a and GATA1-ogm-b paralogons in teleost fish. The GATA1-ogm-a paralogon contained PFKB2, CACNA1S, TIMM17A, PCTK3, ELK4, and TMCC2 ortholog, and lost CAMK1G. The GATA1-ogm-b paralogon, on the other hand, appears to be greatly reduced to a linked PFKB2 and CACNA1S ortholog.

The GATA2 paralogon gave rise to both GATA2a and GATA2b paralogons. The GATA2a paralogon has retained syntenic SLC38A3, PRKCD, ITIH1, ITIH3, SFMBT1, DUSP7, SEC61A1, and GATA2. Meanwhile, the GATA2b paralogon retained SLC38A3, PRKCD, PLXNA1, SEC61A1, GATA2b, and PFKB4 paralogs.

The GATA3 paralogon gave rise to both GATA3 and GATA3-gone missing paralogons. The GATA3 paralog retained GATA3, ITIH2, ITIH5, SFMBT2, PRKCQ, PFKB3, whereas the GATA3-missing retained CAMK1D, TAF3, CHCHD3, and NET1 paralogs.

1.7. Modifications to GATA paralogons in extant fish species (medaka, zebrafish, fugu, stickleback)

There have been multiple modifications from the ancestral fish paralogons to the extant species. The zebrafish GATA1a paralogon contains a duplicated opsin gene, while zebrafish and fugu GATA1a paralogons have lost syntenic TAF4 paralogs. Meanwhile, the GATA1b paralogon has also degenerated, with ITIH5L lost in zebrafish and fugu, PFKB1 lost in zebrafish and stickleback, CACNA1F lost in fugu and translocated along with SLC38AF in zebrafish, while PLXNB3 is mixing in stickleback and medaka.

The GATA2a paralogon in zebrafish and fugu lost SLC38A3 and SFMBT1, in fugu lost PRKCD, in medaka lost ITIH3, in fugu, stickleback, and medaka lost DUSP7, and zebrafish lost and medaka translocated SEC61A1. Meanwhile, the GATA2b paralogon lost or translocated SLC38A3 and PFKB4 in zebrafish and fugu, PRKCD in fugu, and PLXNA1 in zebrafish, fugu, and medaka,.

The GATA3 paralogon lost or translocated ITIH2 in stickleback, and PFKB3 in fugu, stickleback, and medaka, whereas the GATA3-missing paralogon lost or translocated TAF3 and NET1 in zebrafish, CHCHD3 in zebrafish and fugu.

**2. The GATA456 paralogon**

2.1. The ancestral vertebrate GATA456 paralogon (pre R1)

Based upon our analysis, we would predict that the following gene families were likely syntenic in the ancestral GATA456 locus prior to the first round of vertebrate genome duplication (see Figure 5a, Table 3): Oxysterol binding like proteins, (OSBPL1A/2), laminins (LAMA3/5), Cdk5 and Abl enzyme substrates (CABLES1/2), abhydrolase domain containing proteins (ABHD1/3), and sox transcription factors (SOX7/18).

2.2. The ancestral vertebrate GATA4/4-ogm and GATA5/6 paralogons (pre R2)

After the first round of genome duplication two GATA456 paralogons were generated from the initial GATA456 chromosome, which we infer to be the GATA4 paralogon, and the GATA5/6 paralogon. We infer that three losses of three ohnologs have occurred in the GATA4 paralogon, of the OSBPL1A/2, LAMA3/5, and CABLES1/2 paralogs, which would leave three genes (SOX7, GATA4, and ABHD1), while no changes have been identified in the predicted GATA5/6 paralogon, leaving it with SOX18, ABHD3, LAMA3/5, CABLES1/2, OSBPL1A/2, and GATA5/6 orthologs. .

2.3. The ancestral vertebrate GATA4, 4-ogm, 5, and 6 paralogons (post R2)

After the second round of genome duplication, we predict four GATA456 paralogons were generated, including GATA4, GATA4-ogm (which has lost the duplicate GATA4 paralog), GATA5, and GATA6. One loss of a paralog was predicted in both the GATA5 (ABHD3) and GATA6 (SOX18) paralogon. The GATA5 paralogon was then composed of OSBPL2, LAMA5, CABLES2, GATA5, SOX18. The GATA6 paralogon was composed of ABHD3, GATA6, CABLES1, LAMA3, OSBPL1A. We have found no conclusive evidence which would allow us to identify the GATA4-ogm paralogon, possibly due to the degeneration of it or the GATA4 paralogon; therefore we infer that the GATA4 paralogon would contain the same three (SOX7, GATA4, and ABHD1) as the 1R chromosome.

2.4. The ancestral amniote GATA4. 5, and 6 paralogons

We infer the ancestral amniote GATA paralogons from comparisons of chicken, mouse, and human genomic regions. The amniote paralogons appear largely intact, with the only change identified for the GATA4 paralogon, the ABHD1 paralog has been lost in all of the tetrapod genomes, though is found in the GATA4 paralogon in fish. Both the ancestral amniote GATA5 and GATA6 paralogon appears to have all of the predicted ancestral R2 vertebrate paralogs.

2.5. Modification to GATA paralogons in extant tetrapod species (chicken, mouse, human)

There appear to be only minor modifications in the conserved set of paralogons in the tetrapod genomes. As described above, the GATA4 paralogon in all tetrapod genome is lacking the ABHD1 paralog. GATA5 appears to have retained the set of paralogs found in all examined amniote genomes, while two losses (LAMA3, PSMA8) have been identified in the chicken GATA6 paralogon. We have inferred six paralogons, which existed prior to the divergence of acanthopterygiian and ostariophysian fish, based upon our comparisons of five extant species (see below).

2.6. The ancestral fish GATA456 derived paralogons

As fish appear to have undergone a third whole round of genome duplication, for each ancestral chromosome, we would predict to find two paralogons for each ancestral one. If this scenario is correct we would expect two fish paralogons for each the GATA4, GATA5, and GATA6 containing paralogons in the R2 ancestor, giving a total of six teleost GATA456 paralogons, although each additional paralogon appears to have lost the appropriate GATA456 paralog.

The R2 GATA4 paralogon gave rise to the 3R GATA4 and GATA4-ogm paralogons. While the GATA4 paralogon contained the SOX7, GATA4, AND ABHD1 ohnologs, the 3R GATA4-ogm did not retain any identifiable ohnologs, can only be identified by a few syntenic orthologs with the amniote GATA4 and fish GATA4a paralogons (including TDH, MEMR9, and c18ORF13 orthologs).

The R2 GATA5 paralogon gave rise to the 3R GATA5 and GATA5-ogm paralogons. The GATA5 paralogon retained TAF4, OSBPL2, LAMA5, CABLES2, SAMD10, and GATA5 paralogs, while the GATA5-ogm paralogon retained OSBPL2, CABLES2, SAMD10, and SOX18 paralogs. Both contained a number of orthologs shared between teleost fish, both the fish GATA5 and GATA5-ogm paralogons and the single vertebrate GATA5 paralogon.

The R2 GATA6 paralogon gave rise to the 3R GATA6 and GATA6-ogm paralogons. The GATA6 paralogon retains ABHD3, GATA6, CABLES1, LAMA3, OSBPL1a paralogs, while the GATA6-ogm only contained a few syntenic orthologs with the vertebrate GATA6 paralogon (including rbbp8, riok3, and c18orf8).

2.7. Modifications to the GATA paralogons in extant fish species (medaka, zebrafish, fugu, stickleback)

There have been a few modifications from the ancestral fish paralogons to the extant species.

The zebrafish R2 GATA4 paralogon GATA4 paralogon lacks the ABHD1 paralog, the SOX7, GATA4, AND ABHD1 ohnologs, and a few orthologs are found on either chromosome, but for the most part the GATA4 paralogons appear fairly conserved across the fish species.

The GATA5 paralogon appears complete in zebrafish, while both stickleback and medaka GATA5 paralogons have the lost SAMD10, and the medaka GATA5 paralogon is also missing LAMA5 and CABLES2 paralogs. The zebrafish and medaka GATA5-ogm paralogons are missing SAMD10 and SOX18 orthologs, zebrafish is also missing TAF4, and the stickleback GATA5-ogm appears complete. The fugu GATA5 paralogons are unidentifiable; GATA5 is contained on a short scaffold with very few neighboring genes, and we can find no sign of the GATA5-ogm paralogon.

The GATA6 and GATA6-ogm paralogons: The zebrafish GATA6 paralogon lacks ABHD3 and OSBPL1a, whereas the fugu GATA6 paralogon lacks LAMA3 and OSBPL1A, while both the medaka and stickleback GATA6 paralogons appear intact with ABHD3, GATA6, CABLES1, LAMA3, and OSBPL1a paralogs.
